# Physical Properties and Polymorphism of Acrylic Acid-Grafted Poly(1,4-butylene adipate-co-terephthalate)/Organically Modified Layered Double Hydroxide Nanocomposites

**DOI:** 10.3390/polym14030492

**Published:** 2022-01-26

**Authors:** Yun-Ju Chen, Yu-Jia Hung, Ming-Yen Chiang, En-Tze Wang, Tzong-Ming Wu

**Affiliations:** Department of Materials Science and Engineering, National Chung Hsing University, 250 Kuo Kuang Road, Taichung 402, Taiwan; bitcoin2243@gmail.com (Y.-J.C.); tvickytw@yahoo.com.tw (Y.-J.H.); tigercity86@gmail.com (M.-Y.C.); axsz2760089@gmail.com (E.-T.W.)

**Keywords:** poly(1,4-butylene adipate-co-terephthalate), layered double hydroxide (LDH), graft interaction, nanocomposites, polymorphism

## Abstract

Novel and biodegradable acrylic acid-grafted poly(1,4-butylene adipate-co-terephthalate)/organically modified layered double hydroxide (g-PBAT/m-LDH) nanocomposites were synthesized through the polycondensation and transesterification process, with the covalent linkages between the polymer and the inorganic materials. X-ray diffraction and transmission electron microscopy were used to characterize the structure and morphology of the g-PBAT/m-LDH nanocomposites. The experimental results show that the m-LDH was exfoliated and widely distributed in the g-PBAT matrix. The addition of m-LDH into the g-PBAT extensively improved the storage modulus at −90 °C, when compared to that of the pure g-PBAT matrix. The effects of the minor comonomer of the butylene terephthalate (BT) unit and the addition of m-LDH on the crystallization behavior, and the polymorphic crystals of the g-PBAT at numerous crystallization temperatures, were examined, using a differential scanning calorimeter (DSC). The data indicate that the minor comonomer of the BT unit into g-PBAT can significantly change the starting formation temperatures of the α-form and ꞵ-form crystals, while a change in the starting formation temperatures of the α-form and ꞵ-form crystals using the addition of m-LDH into g-PBAT is not evident.

## 1. Introduction

Biodegradable polymers, such as poly(butylene adipate) (PBA), poly(butylene succinate) (PBS), and poly(L-lactic acid) (PLLA), have received a lot of attention owing to their biocompatibility and biodegradability for ecological and environmental benefits [1]. Among these biodegradable polymers, PBA, a semicrystalline polyester, has been extensively investigated in academic studies [2,3]. Two kinds of crystal forms, designated as α- and β-form, using numerous crystallization temperatures (T_cs_), are classified for PBA, for which the α-form crystal is thermodynamically more stable than the β-form crystal. Minke and Blackwell first reported the polymorphic crystals and crystallization behavior of PBA stretched film [2]. Gan et al. found that the crystal structure of PBA was highly sensitive to temperature when the PBA was crystallized at either lower than 28 °C (β-form crystal), or higher than 32 °C (α-form crystal), and a mixture of both α- and β-form crystals was formed between 28 and 32 °C [4,5]. Therefore, the changes in the polymorphic crystals in the PBA caused by various T_cs_ (thermal treatments), and the blending with other polymers, such as poly(benzyl methacrylate), poly(lactide), and poly(vinyl alcohol) (PVA), has been extensively examined [5,6,7,8,9,10,11,12]. For example, Liang et al. investigated the effects of incorporating PVA into PBA on the changes in the α- and β-form crystals [6]. Their results found that the β- and α-form PBA-type crystals were mainly formed at a *Tc* less than 25 °C, and greater than 34 °C, respectively. The change in the formation temperatures probably contributed to the change in the surface free energy for the nucleation of PBA.

Nevertheless, practicable applications of PBA have been restricted owing to its thermal stability, its softness, and its crystallization rate. According to previous studies, the incorporation of inorganic fillers into the PBA as reinforcement materials can improve its hardness, the crystallization rate, and the thermal properties [1,13]. Numerous reinforcing materials, with various sizes and shapes, have been widely utilized in polymer composites [13,14,15]. However, the influence of the reinforcing materials on the polymorphism and crystallization behavior of PBA composites is not frequently stated among these studies. The two-dimensional layered double hydroxides (LDHs), consisting of stacks of positively charged layers of [M^II^_1−*x*_M^III^*_x_*(OH)_2_], where M^II^ and M^III^ are di- and trivalent metal cations comprised of superior electrostatic interactions between the hydroxide layers, make well-dispersed LDHs within the polymer matrix more difficult [16]. In order to overcome the strong electrostatic interactions between the hydroxide layers, organomodifiers have been developed to act as well-dispersed compounds of LDHs. Lately, biocompatible and nontoxic organomodifiers, such as γ-poly(glutamic acid) and oleic acid, have successfully expanded the interlayer spacing of LDHs, and they have received a lot of attention for their biomedical, ecological, and packaging applications [17,18,19]. The polymer chains could be randomly separated and well distributed into the greater interlayer spacing of LDHs to produce the polymer nanocomposites.

In this work, biocompatible and nontoxic dodecanedioic acid is employed as an organomodifier to fabricate the organically modified LDHs by using the anion exchange method (henceforth assigned as “m-LDH”). Three different molar ratios of poly(1,4-butylene adipate-co-terephthalate) (PBAT) were synthesized via transesterification and polycondensation. In order to investigate the polymorphic crystals and morphology of PBAT at various crystallization temperatures (T_cs_), the mole ratios of the butylene terephthalate (BT) units are assigned less than 30 mole percent [20]. Therefore, the fabricated PBAT copolymer will be in the PBA crystalline form. The biodegradable PBAT/m-LDH nanocomposites are prepared through the solution mixing method. To the best of our knowledge, the g-PBAT/m-LDH nanocomposites with covalent linkages between g-PBAT and m-LDH are first reported here. X-ray diffraction and transmission electron microscopy were used to characterize the structure and morphology of the PBAT/m-LDH nanocomposites. The effect of the molar ratio of the BT unit, and the addition of m-LDH on the physical properties and polymorphic crystals of the PBAT/m-LDH nanocomposites at numerous T_cs_, were mainly examined using a differential scanning calorimeter (DSC), and were partially examined using in situ wide-angle X-ray diffraction (WAXD).

## 2. Materials and Methods

### 2.1. Materials

Acrylic acid, adipic acid (AA), azobisisobutyronitrile (AIBN), dodecanedioic acid, and 1-ethyl-3-(3-dimethylaminopropyl)carbodiimide (EDC) were acquired from the Sigma-Aldrich Chemical Company (St. Louis, MO, USA). Aluminum nitrate enneahydrate (Al(NO_3_)_3_·9H_2_O), 1,4-butanediol (BD), dimethylene terephthalate (DMT), and magnesium nitrate hexahydrate (Mg(NO_3_)_2_·6H_2_O) were obtained from the Alfa Aesar Chemical Company (Haverhill, MA, USA). Sodium hydroxide was purchased from the Fluka Chemical Company (Buchs, Switzerland). All chemicals were used without purification.

### 2.2. Preparation of g-PBAT/m-LDH Nanocomposites

The synthesis procedures of g-PBAT and g-PBAT/m-LDH nanocomposites are shown in Figures S1 and S2. The synthesis of magnesium/aluminum layered double hydroxides (LDHs), with a molar ratio of Mg/Al = 2, were prepared via coprecipitation, as reported previously [17,18,19]. An adequate amount of organomodifier, dodecanedioic acid, was mixed with the LDHs, using the anion exchange method, at 90 °C, under nitrogen gas protection, for 24 h, in order to fabricate the organically modified LDHs (m-LDHs).

Three different molar ratios of PBAT were prepared via transesterification and polycondensation. The feed molar ratios of [AA] to [DMT] were 100:0, 90:10, and 80:20, and selected amounts of BD; the resulting products are hereinafter designated as PBA, PBAT-90, and PBAT-80, respectively. In brief, desirable amounts of AA, DMT, BD, and titanium(IV) butoxide as a catalyst were heated and mechanically stirred, at 160 °C for 1 h in a stream of nitrogen gas, and then heated to 190 °C for 2 h to completely distill the water and methanol, and heated to 220 °C for 4 h under vacuum. In order to obtain high-purity PBAT samples, the as-prepared PBAT was dissolved in 100 mL of dichloromethane, and was then precipitated from 1 L of methanol at −10 °C. The above purification process needs to be repeated three times. The obtained polymer ratio, determined using ^1^H-nuclear magnetic resonance, is shown in Table 1. The ^1^H-NMR spectrum of the PBAT-90 copolymer is shown in Figure S3.

The grafting reaction was operated using a mixture of acrylic acid and azobisisobutyronitrile, which were added into the purified and dissolved PBAT in a chloroform solution at 60 °C for 24 h (assigned as “g-PBAT”). Different amounts of g-PBAT, m-LDH, and EDC as a catalyst were individually dissolved in dichloromethane and were then mixed/mechanically stirred for 3 days. The obtained various g-PBAT/m-LDH nanocomposites were washed and dried in vacuum.

### 2.3. Characterization of g-PBAT/m-LDH Nanocomposites

The nuclear magnetic resonance (NMR) spectra were measured by Agilent Technologies DD2 600MHz ^1^H-NMR spectroscopy (Santa Clara, CA, USA), using CDCl_3_ as a solvent standard. The number average molecular weight (M_n_), the weight average molecular weight (M_w_), and the polydispersity (PDI = M_w_/M_n_) of the synthesized materials were determined using gel permeation chromatography (GPC; Waters 717 plus autosampler, Waters Instruments, Rochester, NY, USA). The M_n_, M_w_, and PDI of the synthesized PBAT copolyesters are shown in Table 1. An X-ray diffractometer (Bruker D8, Karlsruhe, Germany), equipped with Ni-filtered Cu Kα radiation, was used for the experiments of the wide-angle X-ray diffraction (WAXD). The measurements of the WAXD were conducted in the range of 2θ = 2–40°, at a scanning rate of 1°/min. The transmission electron microscopy (TEM) images were obtained using a JEOL JEM-2010 (Tokyo, Japan). The thermal degradation of the specimens was obtained using a TGA 2950 thermal gravimetric analyzer (TA Instruments, New Castle, DE, USA). The experiment was carried out from room temperature to 800 °C, under a nitrogen environment, at a heating rate of 10 °C/min. The thermal analysis was analyzed using a Perkin Elmer-PYRIS diamond-differential scanning calorimeter (DSC; Perkin Elmer, Waltham, MA, USA). The specimens were heated to the designed temperatures (T_ds_), which were about 40 °C higher than the melting temperatures of neat PBAT at a heating rate of 10 °C/min, and they were held for 5 min to erase the thermal history. Subsequently, the samples were rapidly cooled to the proposed crystallization temperatures (T_cs_), at a cooling rate of 100 °C/min, and they were held for a period of time until the crystallization was complete. Finally, the samples were heated to the T_ds_ at a rate of 10 °C/min, and the crystalline melting temperatures (T_m_) for the prepared samples were obtained. The mechanical properties of the fabricated materials were operated on a dynamic mechanical analyzer (DMA8000, Perkin Elmer, Waltham, MA, USA), from −90 to 30 °C, at a 2 °C/min heating rate, and a constant frequency of 1 Hz.

## 3. Results

### 3.1. Structures and Polymorphisms of Various g-PBAT Copolymers

**Figure 1:**
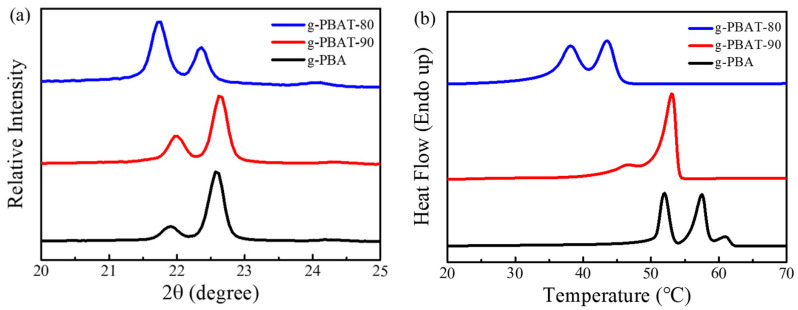
(**a**) WAXD patterns; and (**b**) DSC heating curves of g-PBA, g-PBAT-90, and g-PBAT-80 polyesters.

Figure 1a reveals the WAXD diffraction curves of the g-PBA homopolymer, and the g-PBAT-90 and g-PBAT-80 copolyesters. For the g-PBAT copolymers containing two crystallizable comonomer units, the crystalline structure of the g-PBAT is determined by the composition of the copolyesters. In order to investigate the structures and polymorphisms of the various g-PBAT copolymers, the mole ratios of the BT units were designed as 10 mole and 20 mol percentages in order to obtain the PBA crystalline form. As revealed in this figure, the diffraction profiles of all of the polymers are almost the same. The two strong diffraction peaks at 2θ = 21.8° and 22.5°, and a weak diffraction peak at 2θ = 24.1°, are attributed to the β-crystal of the PBA [3,4]. This data reveals that the prepared g-PBAT-90 and g-PBAT-80 copolymers contain the crystalline structure of PBA. The detailed procedure of the crystal form determination is listed in SI. It can be seen that the peak positions and intensities were slightly shifted in the g-PBAT-90 and g-PBAT-80 copolymers, which might be attributed to the fact that the grafting interaction of acrylic acid onto PBAT induces more steric hindrance to change the polymer chain packing during crystallization. Figure 1b shows the DSC heating curves of g-PBA, g-PBAT-90, and g-PBAT-80. The melting profiles of all the polymers contain multiple melting behaviors. It is clear that the addition of the crystallizable comonomer BT units into PBA might reduce their melting temperatures. The melting temperatures of g-PBA, g-PBAT-90, and g-PBAT-80, determined by DSC, are 52.0 °C, 46.5 °C, and 38.1 °C, respectively. These results reveal that the grafting interaction onto the PBAT slightly changed their melting temperatures, perhaps indicating that the incorporation of acrylic acid into the PBAT polymer backbone causes more steric hindrance, reducing the transportation ability of the polymer chains during crystallization, and subsequently increasing their T_ms_.

**Figure 2:**
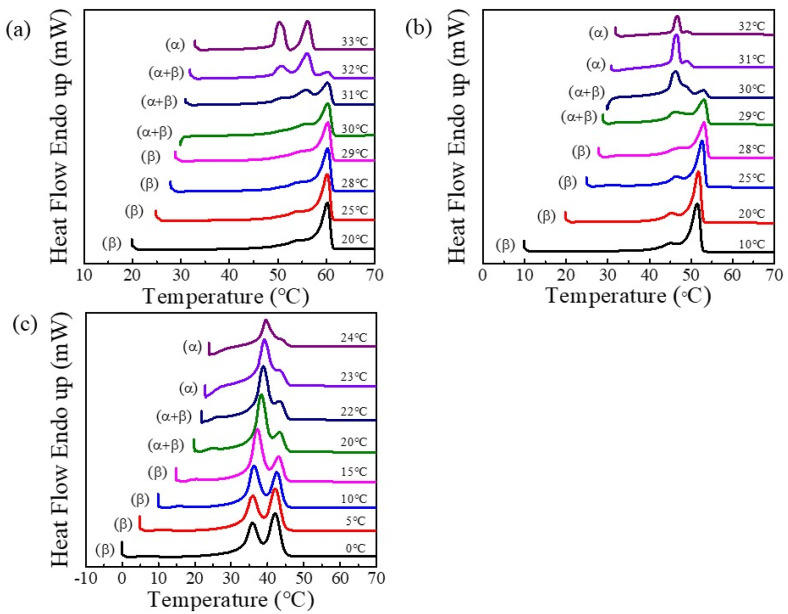
DSC heating curves of (**a**) g-PBA, (**b**) g-PBAT-90, and (**c**) g-PBAT-80, crystallized at different temperatures, as indicated on traces.

**Figure 3:**
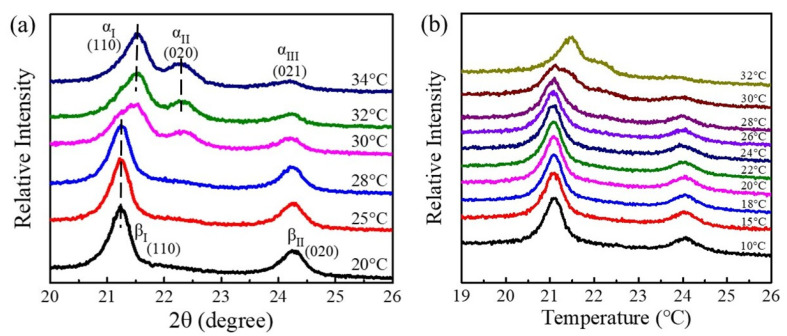
In situ WAXD patterns of (**a**) g-PBA, and (**b**) g-PBAT-90, crystallized at different temperatures, as indicated on traces.

The polymorphism behaviors and crystalline phases of g-PBA, g-PBAT-90, and g-PBAT-80 were investigated. Figure 2 illustrates the DSC heating scans of g-PBA, g-PBAT-90, and g-PBAT-80, which were isothermally crystallized at various crystallization temperatures. Because the fabricated g-PBAT copolymers contain the mole ratios of BT units less than 30 mole percent, the fabricated g-PBAT copolymers will be in the PBA crystalline form. These results indicate that the fabricated materials with polymorphic crystal structures showed different melting behaviors. For g-PBA, both the α- and β-crystals show double melting peaks, while the mixed crystal shows triple melting peaks. These experimental results reveal that the melting behavior of g-PBA at or below 29 °C represents β-crystal formation, whereas those of crystallization temperatures at or above 33 °C represent α-crystal formation. The melting behavior of g-PBAT-90 is similar to that of g-PBA, in which crystallization temperatures at or below 28 °C represent β-crystal formation, and crystallization temperatures at or above 31 °C represent α-crystal formation. Because the melting temperature of PBAT-80 is about 38 °C, the melting profile of g-PBAT-80 is completely different from those of g-PBA and g-PBAT-90. Both of the double melting peaks of the α- and β-crystals were clear shifted towards the lower temperature. These DSC data reveal that the melting behavior of g-PBAT-80 at or below 15 °C represents β-crystal formation, whereas those of crystallization temperatures at or above 23 °C represent α-crystal formation. In order to further verify the crystal formation, an in situ WAXD was applied to these fabricated materials. The WAXD data of g-PBA, g-PBAT-90, and g-PBAT-80 are shown in Figure 3. The diffraction peaks assigned to the α- or β-crystal forms are noticeably characterized on the g-PBA diffraction profiles. When the isothermal crystallization temperatures are equal to or below 28 °C, two strong diffraction peaks are observed at 2θ = 21.3° and 24.4°, labeled as the βI and βII peaks, respectively. Conversely, as the crystallization temperatures reach 32 °C or above, there are two strong diffraction peaks at 2θ = 21.8° and 22.5° (αI and αII), and a weak diffraction peak at 2θ = 24.1° (αIII), which were found to be located at entirely different positions than those at or below 28 °C, suggesting that the crystal structure of g-PBA at or above 32 °C is extremely different from that at or below 28 °C. These results reveal that the diffraction profiles of isothermally crystallized g-PBA at or below 28 °C represent β-crystal formation, whereas those of crystallization temperatures at or above 32 °C represent α-crystal formation. The diffraction peaks of the isothermally crystallized g-PBA, ranging from 28 to 32 °C, contain all of the diffraction peaks, suggesting that a mixed α- and β-crystal formation is obtained. The in situ WAXD pattern of g-PBAT-90 is close to that of g-PBA. The experimental results show β-crystal formation under the in situ isothermal crystallization at or below 26 °C, while the α-crystal formation was observed at 32 °C. In the range between 26 and 30 °C, a mixed of α- and β-crystal formation is obtained. This result is slightly different from the data of the DSC isothermal crystallization because of the open-air environment for the in situ WAXD experiments. Because the changes in the α- and β-crystals for PBAT-80 are in the range from about 15 to 23 °C, the in situ WAXD experiment cannot be operated at lower temperatures. Therefore, DSC isothermal crystallization was applied for the further crystalline phases and polymorphic behaviors of the g-PBAT/m-LDH nanocomposites.

### 3.2. Morphologies, Physical Properties, and Polymorphisms of Various g-PBAT/m-LDH Nanocomposites

**Figure 4:**
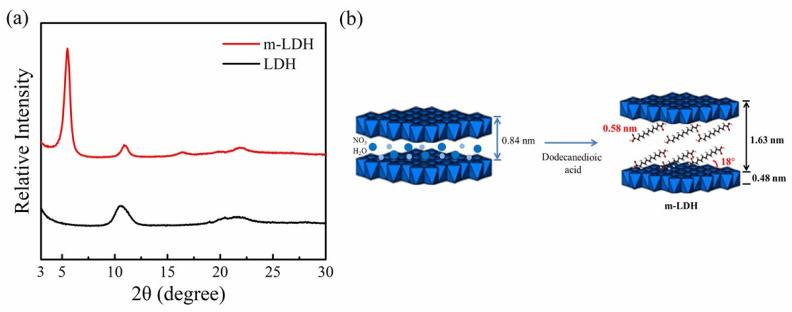
(**a**) X-ray diffraction patterns for LDH and m-LDH. (**b**) Schematic representation of the probable arrangement for dodecanedioic acid-modified LDH (m-LDH).

Figure 4 shows the WAXD profiles of the LDHs and m-LDHs, prepared using the coprecipitation method. The data of the LDHs clearly show the formation of a well-stacked lamellae structure [19,21,22,23]. According to the Bragg’s equation, the interlayer spacing of the hydroxide layers, obtained from the diffraction peak at 2θ = 10.6°, was determined to be 8.4 Å. The diffraction data of the m-LDHs indicate that the main diffraction peak was changed to a lower angle with the addition of dodecanedioic acid as an organomodifier, which reveals that the interlayer spacing of the hydroxide layers obtained from the diffraction peak at 2θ = 5.4° was enlarged to 16.3 Å after the anionic exchange of dodecanedioic acid. These results imply that the dodecanedioic acid was effectively intercalated and exchanged into the interlayer spacing of the LDHs. Therefore, a probable arrangement of dodecanedioic acid-modified LDH is schematically represented in Figure 4b.

**Figure 5:**
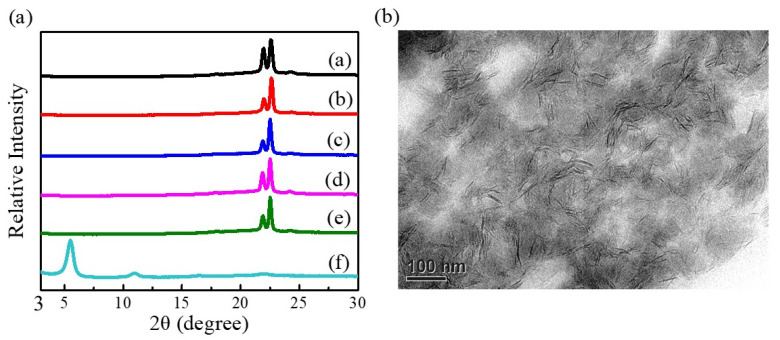
(**a**) WAXD patterns of g-PBAT-90 and m-LDHs, and various weight ratios of g-PBAT-90/m-LDH nanocomposites. (**b**) TEM micrographs of 5 wt% g-PBAT-90/m-LDH nanocomposites.

The X-ray diffraction data of the fabricated g-PBAT-90/m-LDH nanocomposites are shown in Figure 5a. For comparison, the X-ray diffraction data of the m-LDH is also presented in this figure. The main diffraction peak corresponding to the m-LDH disappeared in all of the diffraction profiles of the g-PBAT-90/m-LDH nanocomposites with various loadings of the m-LDH content. This observation can probably be attributed to the formation of an exfoliated structure, in which the interlayer distance was not large enough and cannot be detected by an X-ray instrument. This result suggests that the molecular chains of g-PBAT-90 were well inserted and widely dispersed in the m-LDH galleries, even though a 5 wt% of m-LDH was incorporated. Similar results were also obtained for the g-PBA/m-LDH and g-PBAT-80/m-LDH nanocomposites. While the X-ray diffraction data exhibits a partial representation of the distribution of the LDHs, the complete confirmation of the exfoliated morphology for g-PBAT/m-LDH requires microscopic examination. Figure 5b shows the TEM micrograph of a 5 wt% g-PBAT-90/m-LDH nanocomposite. This result reveals that the original stacked lamellar structure of the m-LDH can be modified to form the disorderly dispersed morphology in the g-PBAT-90 matrix. Similar TEM observations are also obtained for the g-PBA/m-LDH and g-PBAT-80/m-LDH nanocomposites. Both the X-ray diffraction and the TEM results exhibit that the hydroxide layers are randomly distributed and exfoliated in the g-PBAT matrix. Therefore, the g-PBAT/m-LDH nanocomposites with exfoliated conformation were well prepared using the solution mixing process.

**Figure 6:**
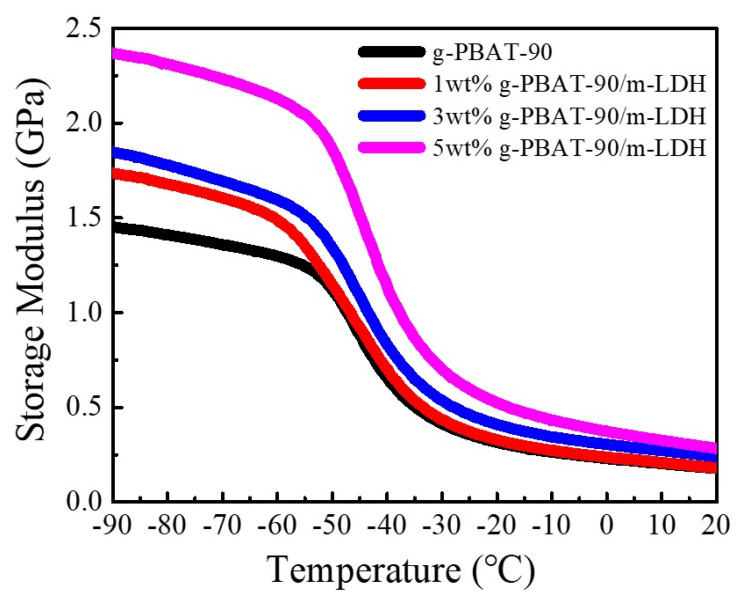
Dependences of the storage moduli on temperatures of g-PBAT-90/m-LDH nanocomposites.

**Figure 7:**
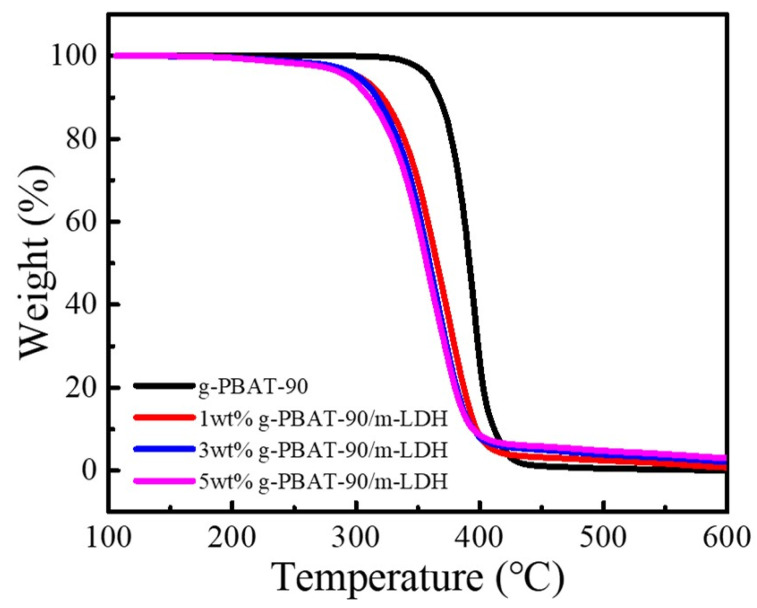
TGA profiles of g-PBAT-90 and various weight ratios of g-PBAT-90/m-LDH nanocomposites.

The changes in the storage modulus, E*’*, as a function of the temperature, ranging from −90 to 20 °C for the g-PBAT-90/m-LDH nanocomposites, are shown in Figure 6. These results reveal that the E*’* of g-PBAT-90 is about 1.45 GPa at −90 °C, and that it decreases with increasing temperature. This result suggests that the molecular mobility of g-PBAT-90 at the lower temperature of a glassy state is insufficient. As the temperature increases above the glass transition temperature, the thermal energy can almost overcome the potential energy barriers of the molecular mobilities. The E’ of the g-PBAT-90/m-LDH nanocomposites at −90 °C increased as the loading of the m-LDHs increased. A similar tendency of the E*’* was also observed for the g-PBA/m-LDH and g-PBAT-80/m-LDH nanocomposites. Detailed storage moduli for all of the nanocomposites are also illustrated in Table 2. The addition of the stiff m-LDH and its covalent bonds with g-PBAT may cause the enhancement in the rigidity of the g-PBAT polymer matrix, which leads to the improvement of the E*’*. The effect of m-LDH on the thermal stabilities of the numerous g-PBAT polymer matrices was determined using TGA analysis. Figure 7 reveals the TGA heating profiles of the g-PBAT-90/m-LDH nanocomposites. A similar tendency to the TGA profile was also observed for the g-PBA/m-LDH and g-PBAT-80/m-LDH nanocomposites. Table 2 shows the degradation temperatures found from these experimental curves. The thermal degradation temperature of neat g-PBAT-80 is slightly higher than those of g-PBA and g-PBAT-90, indicating that neat g-PBAT-80 presents the better thermal stability among these synthesized polymers. This phenomenon is attributed to the existence of more rigid BT units in the g-PBAT-80 matrix, which can result in an increase in the thermal stability. However, the degradation temperatures of the g-PBAT/m-LDH nanocomposites decreased with increasing loadings of m-LDH. This occurrence is attributed to the existence of the lower thermal stability of the dodecanedioic acid serving as an organomodifier on the surface of the m-LDH, causing the decrease in the thermal stability for the g-PBAT/m-LDH nanocomposites.

**Figure 8:**
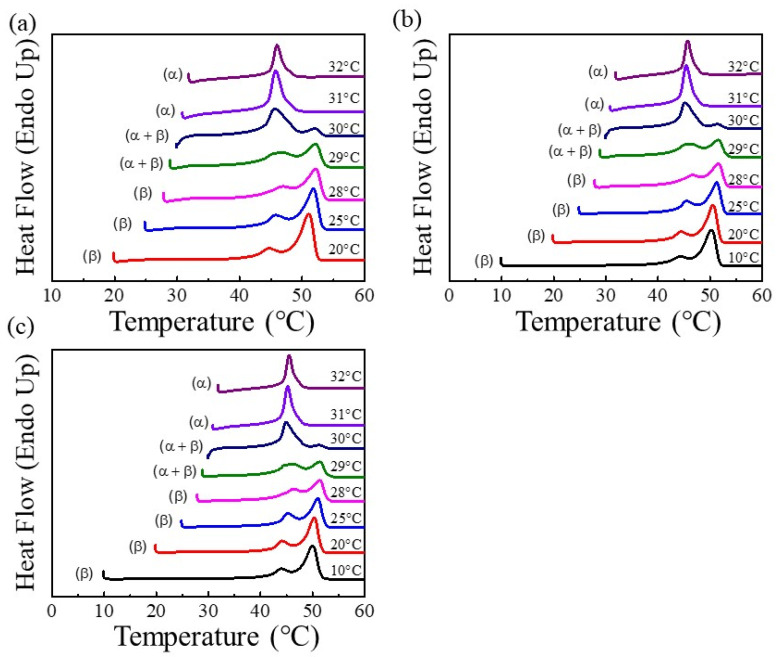
DSC heating curves of (**a**) 1 wt% g-PBAT-90/m-LDH, (**b**) 3 wt% g-PBAT-90/m-LDH, and (**c**) 5 wt% g-PBAT-90/m-LDH nanocomposites, crystallized at different temperatures, as indicated on traces.

The crystalline phases and polymorphic behaviors of the g-PBAT/m-LDH nanocomposites were examined using a DSC. Figure 8 illustrates the DSC heating scans of the g-PBAT-90/m-LDH nanocomposites, which were isothermally crystallized at numerous crystallization temperatures. These results indicate that the fabricated materials with polymorphic crystal structures were strongly affected by their isothermal crystallization temperatures. For the g-PBAT-90/m-LDH nanocomposites, the various α- or β-crystal formations also presented in this figure are apparently dependent on the T_cs_. The incorporation of m-LDH can slightly change the formation temperatures of the α- or β-crystal formation crystals, and the increasing loading of m-LDH plays less of a role in the formation temperature changes of the α- or β-crystal formation crystals. This phenomenon is attributed to the formation of covalent bonds between the m-LDH and g-PBAT to form a homogeneous structure, which induces less change in the starting formation temperatures of the α-form and β-form crystals with the increasing loading of m-LDH. The detailed crystal formations for all of the nanocomposites are also illustrated in Table 3.

## 4. Conclusions

Biodegradable g-PBAT/m-LDH nanocomposites are synthesized through the polycondensation and transesterification process, with the covalent linkages between the polymer and the inorganic materials. Both the X-ray diffraction and the TEM results show that the m-LDH is exfoliated and widely distributed in the g-PBAT matrix. The incorporation of 5 wt% of m-LDH into the g-PBAT extensively enhanced the storage modulus at −90 °C, more than 70%, as compared to the pure g-PBAT matrix. The additional 20 mole percent BT unit into PBA can significantly reduce the formation temperature of the β- and α-form crystals, from 28 °C and 32 °C, to 15 °C and 23 °C, respectively. The results might be attributed to the incorporation of the minor BT comonomer into the PBA crystal, inducing the steric hindrance for the PBA polymer chain packing. The change in the starting formation temperatures of the α-form and β-form crystals for the g-PBAT/m-LDH nanocomposites is not evident. This phenomenon is attributed to the formation of covalent bonds between the m-LDH and g-PBAT to form a homogeneous structure.

## Figures and Tables

**Table 1:** Compositions and molecular weights of synthesized polyesters.

Polymer	Feed Ratio [AA]/[DMT] (mol %)	Polymer Ratio ^a^ [AA]/[DMT] (mol %)	M_w_ (g/mol) ×10^4^	M_n_ (g/mol) ×10^4^	PDI	T_c_ (°C)	T_m_ (°C)
PBA	100/0	100:0	4.52	3.14	1.44	29.9	51.25
PBAT-90	90/10	90.6:9.40	4.53	2.62	1.73	19.79	44.75
PBAT-80	80/20	79.4:20.6	4.10	2.37	1.72	5.57	37.1

^a^ Composition measured by ^1^H–NMR.

**Table 2:** Thermal degradation temperatures at 10% and 50% weight losses and storage modulus in bending mode, at −90 °C, of the various g-PBAT/m-LDH nanocomposites.

Sample	^a^T_d_^10%^ (°C)	^b^T_d_^50%^ (°C)	^c^E*’* (GPa)
g-PBA	351.9	388.1	1.65
1 wt% g-PBA/m-LDH	330.8	372.2	2.11
3 wt% g-PBA/m-LDH	309.8	359.3	2.55
5 wt% g-PBA/m-LDH	306.9	357.5	2.92
g-PBAT90	366.6	392.3	1.45
1 wt% g-PBAT-90/m-LDH	324.4	366.5	1.73
3 wt% g-PBAT-90/m-LDH	316.5	360.2	1.86
5 wt% g-PBAT-90/m-LDH	311.2	357.7	2.37
g-PBAT80	367.8	408.4	0.85
1 wt% g-PBAT-80/m-LDH	331.7	375.3	1.06
3 wt% g-PBAT-80/m-LDH	320.2	367.0	1.29
5 wt% g-PBAT-80/m-LDH	316.7	361.2	1.45

^a^T_d_^10%^: thermal degradation temperature at 10% weight loss; ^b^T_d_^50%^: thermal degradation temperature at 50% weight loss; ^c^E’: storage modulus in bending mode.

**Table 3:** Polymorphisms of the various g-PBAT/m-LDH nanocomposites were measured using DSC at various crystallization temperatures.

Sample	T_c_ (°C)	Polymorphism
	29	β-form
	30	α- and β-form
g-PBA	32	α- and β-form
	33	α-form
	28	β-form
	29	α- and β-form
1 wt% g-PBA/m-LDH	31	α- and β-form
	32	α-form
	27	β-form
	28	α- and β-form
3 wt% g-PBA/m-LDH	31	α- and β-form
	32	α-form
	27	β-form
	28	α- and β-form
5 wt% g-PBA/m-LDH	31	α- and β-form
	32	α-form
	28	β-form
	29	α- and β-form
g-PBAT-90	30	α- and β-form
	31	α-form
	28	β-form
	29	α- and β-form
1 wt% g-PBAT-90/m-LDH	30	α- and β-form
	31	α-form
	28	β-form
	29	α- and β-form
3 wt% g-PBAT-90/m-LDH	30	α- and β-form
	31	α-form
	28	β-form
	29	α- and β-form
5 wt% g-PBAT-90/m-LDH	30	α- and β-form
	31	α-form
	15	β-form
	20	α- and β-form
g-PBAT-80	22	α- and β-form
	23	α-form
	15	β-form
	20	α- and β-form
1 wt% g-PBAT-80/m-LDH	23	α- and β-form
	24	α-form
	15	β-form
	20	α- and β-form
3 wt% g-PBAT-80/m-LDH	23	α- and β-form
	24	α-form
	15	β-form
	20	α- and β-form
5 wt% g-PBAT-80/m-LDH	23	α- and β-form
	24	α-form

## Supplementary Materials

The following supporting information can be downloaded at https://www.mdpi.com/article/10.3390/polym14030492/s1, Figure S1: The synthesis procedure of g-PBAT. Figure S2: The synthesis procedure of g-PBAT/m-LDH nanocomposites. Figure S3: 1H-NMR spectrum of the PBAT-90 copolymer.
