# Supplementary material for: Physical Properties and Polymorphism of Acrylic Acid-Grafted Poly(1,4-butylene adipate-co-terephthalate)/Organically Modified Layered Double Hydroxide Nanocomposites
Source: Polymers (Basel). 2022 Jan 26;14(3):492. doi: 10.3390/polym14030492 (PMC8839520; doi:10.3390/polym14030492)
Supplement: Supplementary file 1 [file polymers-14-00492-s001.zip › polymers-1550995-supplementary.pdf]

Supplementary information

Physical Properties and Polymorphism of Acrylic Acid-Grafted Poly(1,4-butylene adipate-co-terephthalate)/Organically Modified Layered Double Hydroxide Nanocomposites

Yun-Ju Chen, Yu-Jia Hung, Ming-Yen Chiang, En-Tze Wang and Tzong-Ming Wu *

Department of Materials Science and Engineering, National Chung Hsing University, 250 Kuo Kuang Road, Taichung 402, Taiwan; bitcoin2243@gmail.com (Y.-J.C.); tvickytw@yahoo.com.tw (Y.-J.H.); tigercity86@gmail.com (M.-Y.C.); axsz2760089@gmail.com (E.-T.W.)

* Correspondence: tmwu@dragon.nchu.edu.tw; Tel.: +886-4-22840500 (ext. 806)

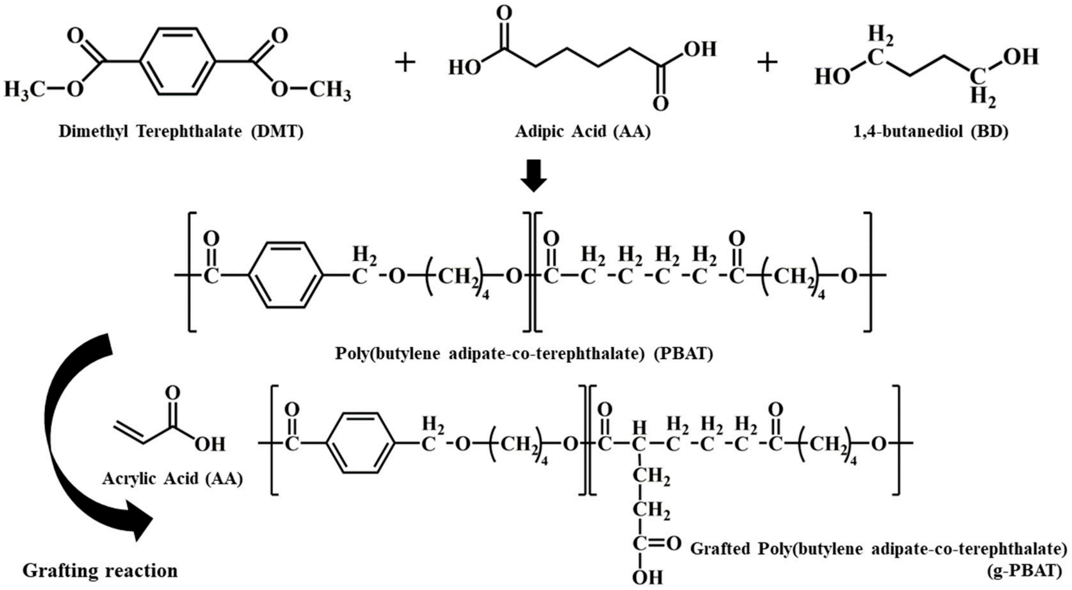

Figure S1: The synthesis procedure of g-PBAT.

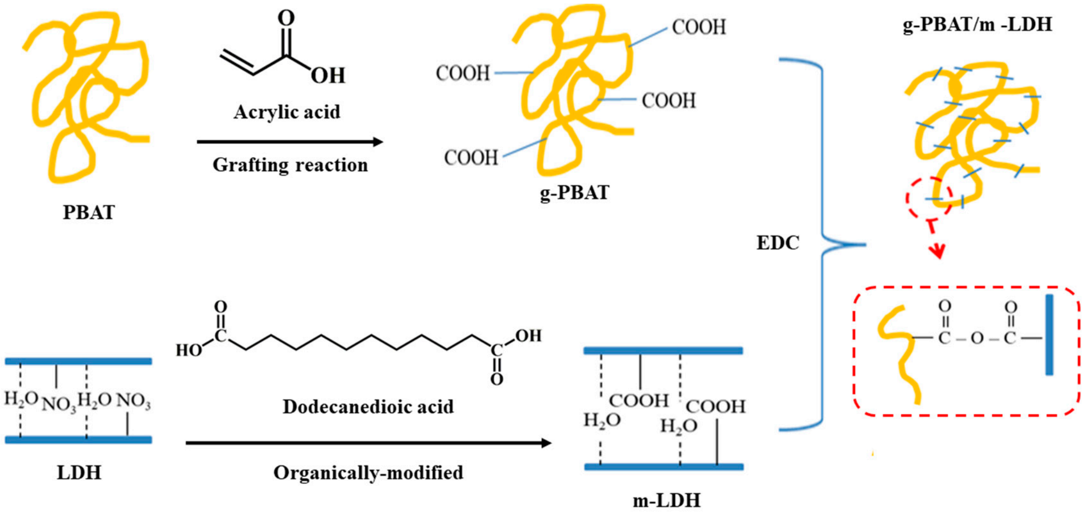

Figure S2: The synthesis procedure of g-PBAT/m-LDH nanocomposites.

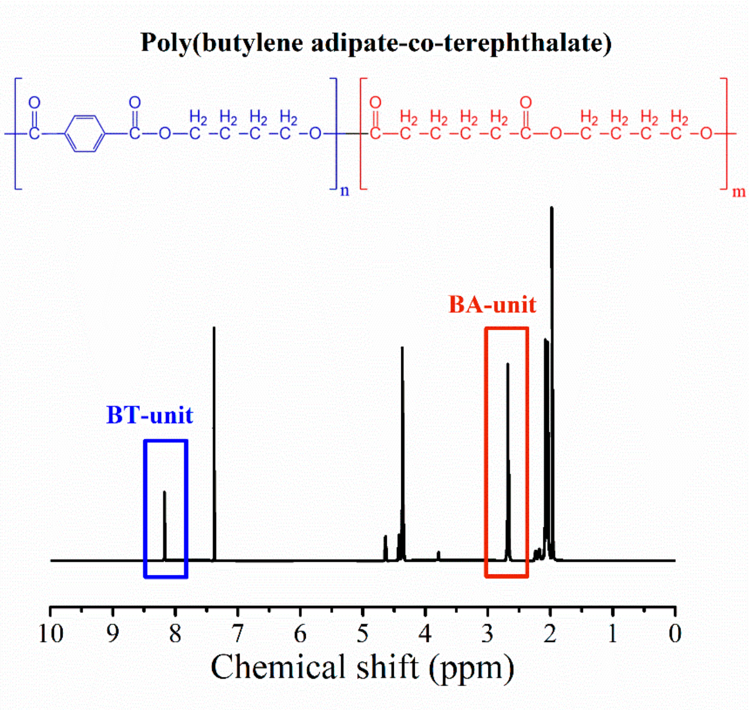

Figure S3: ^{1}H-NMR spectrum of the PBAT-90 copolymer.

The detailed procedure of crystal form determination is listed as following: The diffraction peaks of PBT should be observed at 2θ = 16.3°, 17.4°, 20.6°, 23.3°, and 25.3°. No diffraction peaks were observed at 2θ = 20.6°, 23.3°, and 25.3° as shown in Figure 1a. The diffraction peaks observed in Figure 1a were at 2θ = 21.8°, 22.5° and 24.1°, which were attributed to *β*-crystal of PBA.
